# Effects of demographic variables on mathematics self-esteem, test anxiety, and achievement in adolescents: a Bayesian beta regression analysis

**DOI:** 10.3389/fpsyg.2026.1712257

**Published:** 2026-02-04

**Authors:** Ngozi M. Nwoye, Moeketsi Mosia, Felix O. Egara, Tshele Moloi, Moses Basitere

**Affiliations:** 1 University of Nigeria, Nsukka, Nigeria; 2 University of the Free State, Bloemfontein, South Africa; 3 Sol Plaatje University, Kimberley, South Africa; 4 University of Cape Town, Rondebosch, South Africa

**Keywords:** adolescents, Bayesian analysis, hierarchical modelling, mathematics education, self-esteem, test anxiety

## Abstract

**Background:**

Mathematics achievement is shaped not only by cognitive skills but also by psychological factors such as self-esteem and test anxiety. Demographic characteristics, including gender, age, and school location, may further moderate these relationships, yet limited research has explored these dynamics in diverse educational contexts. Situated in Nigeria, this study examined how mathematics self-esteem and test anxiety predict mathematics achievement among adolescents, while considering the moderating role of demographic variables.

**Methods:**

A cross-sectional design was employed with data collected from 329 secondary school students. Validated instruments measured mathematics self-esteem, mathematics test anxiety, and mathematics achievement. Bayesian beta regression was used to analyse the relationships among psychological and demographic variables, offering a nuanced understanding beyond traditional statistical approaches.

**Results:**

Findings revealed that mathematics self-esteem positively predicted achievement, while test anxiety was negatively associated with performance. Older male students reported lower self-esteem, rural students reported higher test anxiety, and rural students demonstrated higher achievement despite elevated anxiety. Two-way interaction effects involving gender, age, and location accounted for additional variance in mathematics self-esteem and achievement.

**Conclusion:**

The results highlight the complex interplay between psychological and demographic variables in shaping mathematics outcomes. Strengthening students’ self-esteem and addressing test anxiety are essential for promoting equitable learning environments. The findings underscore the importance of demographic context, pointing to the need for targeted interventions and policies that support resilience, reduce anxiety, and enhance adolescent mathematics achievement.

## Introduction

Mathematics achievement is widely recognised as a critical predictor of students’ future educational and career opportunities, particularly in science, technology, engineering, and mathematics (STEM) fields (Egara and Mosimege, 2023, 2024a). Strong mathematics skills are associated with higher levels of problem-solving, employability, and socio-economic mobility, while poor performance in mathematics can limit access to advanced studies and competitive careers (Mosia and Egara, 2025; Egara and Mosimege, 2024b; Rozgonjuk et al., 2020). Despite its importance, mathematics achievement remains uneven across populations, with many adolescents experiencing persistent difficulties. These challenges are not solely academic; they are closely tied to students’ emotional and psychological experiences of learning mathematics (Egara and Mosimege, 2025; Pekrun et al., 2017).

This study was conducted in Nigeria, where mathematics occupies a central position in the secondary school curriculum. The Nigerian education system follows a 6–3–3–4 structure, comprising 6 years of primary education, 3 years of junior secondary school, and 3 years of senior secondary school (Federal Republic of Nigeria, 2013; UNESCO International Institute for Capacity Building in Africa, 2023; Oribhabor, 2020). Mathematics is a compulsory subject throughout secondary education and a core requirement for progression to tertiary education (Federal Republic of Nigeria, 2013). At the end of senior secondary schooling, students sit for high-stakes external examinations, most notably the West African Senior School Certificate Examination (WASSCE), in which performance in mathematics plays a decisive role in certification and access to post-secondary opportunities (West African Examinations Council, 2022).

Educational experiences in Nigeria also vary across geographic contexts. Prior research and national reports indicate that urban schools generally have greater access to instructional resources, specialised teachers, and infrastructure, whereas rural schools often face structural constraints related to staffing, facilities, and learning materials (World Bank, 2020). Some studies further suggest that rural schools may, in certain contexts, offer relatively smaller class sizes and closer teacher–student relationships, although these characteristics vary widely across regions and school systems (Arnold et al., 2005). Such contextual differences are not uniform but may shape students’ mathematics self-esteem, anxiety, and achievement in different ways, underscoring the importance of examining affective and academic outcomes within specific educational settings.

One of the most widely studied emotional factors influencing mathematics performance is mathematics anxiety, which refers to feelings of tension, fear, or apprehension when engaging with mathematical tasks (Ashcraft and Krause, 2007; Egara and Mosimege, 2024a; Nzeadibe et al., 2023; Mosimege et al., 2024). High levels of mathematics anxiety can disrupt working memory, hinder problem-solving efficiency, and lead to avoidance of challenging tasks, ultimately lowering achievement (Namkung et al., 2019; Van Mier et al., 2019).

It is important to distinguish between several related but theoretically distinct motivational constructs that are often conflated in mathematics education research, namely self-esteem, self-concept, and self-efficacy. Self-esteem refers to an individual’s evaluative judgement of self-worth, while academic self-concept reflects beliefs about competence within a specific academic domain, such as mathematics, shaped by prior achievement, feedback, and social comparison processes (Marsh and Craven, 2006). Self-efficacy, as conceptualised within Social Cognitive Theory, refers to task-specific beliefs about one’s capability to perform particular actions successfully (Bandura, 1997).

Although these constructs are theoretically distinct, the present study retains the term *mathematics self-esteem* to reflect the operational definition and wording of the instrument used. The Mathematics Self-Esteem Scale employed in this study assesses students’ perceived competence, confidence, and evaluative feelings toward mathematics, which align with domain-specific academic self-esteem as conceptualised in prior educational research (Harter, 2012; Marsh and Craven, 2006). To avoid construct ambiguity, the term *mathematics self-esteem* is used consistently throughout this article to refer to this domain-specific evaluative belief rather than global self-worth.

Students with a more positive mathematics self-esteem are more likely to persevere with challenging mathematical tasks, adopt adaptive learning strategies, and attain higher levels of achievement (Acosta-Gonzaga, 2023; Okeke et al., 2025). Conversely, weaker mathematics self-esteem may heighten vulnerability to mathematics anxiety and disengagement, thereby undermining performance (Ramirez et al., 2018).

The relationship between mathematics anxiety, self-esteem, and achievement is not isolated but shaped by demographic factors such as gender, age, and educational context (Wong and Ramo, 2025). Gender differences are particularly well documented, with numerous studies showing that girls often report higher levels of mathematics anxiety and lower self-concept compared to boys, even when their performance is similar or higher (Goetz et al., 2013; Hill et al., 2016). Similarly, developmental research suggests that adolescence is a critical period during which mathematics-related beliefs and emotions change significantly (Larsen and Luna, 2018). Transitions from lower to upper secondary schooling are often accompanied by increased academic demands, social comparisons, and identity development, which can heighten vulnerability to anxiety and fluctuations in self-esteem (Eccles and Wigfield, 2020). These dynamics highlight the need to consider developmental timing when examining psychological factors related to mathematics achievement.

In addition to individual factors, contextual influences such as geographic location also play a key role in shaping students’ mathematical experiences. Students in rural areas often face unique challenges, including limited access to advanced coursework, fewer specialised teachers, and reduced educational resources (Maniram, 2023). However, rural schools may also offer advantages, such as smaller class sizes and closer teacher-student relationships, which can foster supportive learning environments. These mixed influences suggest that location may interact with psychological variables, producing distinct patterns of self-esteem, anxiety, and achievement across rural and urban settings.

Although prior studies have explored mathematics anxiety, self-esteem, and achievement, significant gaps remain. Much of the existing research has been conducted in Western, urban, or high-resource contexts, leaving a limited understanding of how these relationships manifest in diverse cultural and socio-economic settings (Namkung et al., 2019; Ramirez et al., 2018). Evidence from large-scale international assessments such as the Trends in International Mathematics and Science Study (TIMSS) demonstrates substantial cross-national variation not only in mathematics achievement but also in students’ attitudes toward mathematics, including confidence, anxiety, and self-beliefs (Mullis et al., 2020). Research focusing on developing regions further highlights how socio-cognitive and affective barriers, such as low self-esteem, test anxiety, and limited perceived control, constrain mathematics learning opportunities in low-resource educational systems (Hunt et al., 2021).

Moreover, most studies have examined main effects in isolation rather than investigating how demographic factors such as gender, age, and location interact to influence psychological and academic outcomes. Additionally, traditional frequentist statistical approaches have limited capacity to capture the complexity of these interactions and the uncertainty inherent in educational data (Gabry et al., 2019). To address these gaps, the present study examines how demographic variables, specifically gender, age, and geographic location, shape mathematics self-esteem, anxiety, and achievement among adolescents. By applying Bayesian beta regression, this study provides a more comprehensive analysis of how these factors interact, offering insights that can inform targeted interventions to reduce mathematics anxiety, strengthen self-esteem, and ultimately improve mathematics achievement.

### Theoretical framework

This study is grounded in two interrelated theories that provide a comprehensive lens for understanding the psychological and contextual factors influencing adolescents’ mathematics achievement: Pekrun’s Control-Value Theory of Achievement Emotions and Bandura’s Social Cognitive Theory. Together, these frameworks explain how students’ beliefs about their mathematical competence interact with their emotional experiences and environmental contexts to shape motivation and performance.

The Control-Value Theory of Achievement Emotions (CVT), first articulated by Pekrun (2006) and later expanded by Pekrun et al. (2017), posits that academic emotions are driven by two key cognitive appraisals: perceived control and subjective value. *Perceived control* refers to the degree to which learners believe they can influence outcomes through their efforts and strategies. At the same time, *subjective value* represents the importance they attach to the academic domain or task. CVT suggests that negative emotions, such as mathematics anxiety, emerge when students place a high value on success in mathematics but perceive limited control over their ability to perform well. For instance, a student who views mathematics as crucial for future aspirations but doubts their competence is likely to experience heightened anxiety, which disrupts working memory and problem-solving efficiency (Pekrun et al., 2002). Conversely, when students perceive high control and value, they are more likely to experience positive emotions such as enjoyment or pride, fostering deeper engagement and persistence.

Within the context of this study, CVT provides a theoretical foundation for understanding the dual roles of mathematics self-esteem and anxiety. In this study, mathematics self-esteem is conceptualised as a domain-specific evaluative belief about competence and confidence in mathematics, closely aligned with academic self-concept rather than global self-worth. Mathematics self-esteem reflects students’ evaluative sense of competence and confidence in mathematics and is conceptually linked to the control component of CVT. Students with higher self-esteem are expected to perceive greater control over their performance, thereby experiencing lower anxiety levels and demonstrating higher achievement levels. In contrast, low self-esteem is associated with diminished perceptions of control, which amplifies anxiety and undermines performance. This framework is particularly relevant for interpreting demographic interactions: for example, girls and older adolescents may encounter stereotype threats or heightened peer comparisons that reduce perceived control, increasing anxiety and decreasing achievement, even when their objective competence is equivalent to that of their peers. By emphasising the interplay between control and value, CVT helps explain why certain demographic groups are more vulnerable to negative emotions and lower mathematics outcomes.

Complementing this perspective, Social Cognitive Theory (SCT), developed by Bandura (1986, 1997), offers a broader framework for understanding how individual beliefs, behaviours, and environmental contexts interact in reciprocal determinism. A central construct of SCT is self-efficacy, defined as individuals’ beliefs in their capacity to organise and execute the actions necessary to achieve desired outcomes. Bandura identified four primary sources of self-efficacy: mastery experiences, vicarious experiences, social persuasion, and physiological and emotional states. In educational contexts, self-efficacy influences students’ motivation, persistence, and resilience, particularly when facing challenging tasks such as mathematics problem-solving (Usher and Pajares, 2008).

Although self-efficacy and mathematics self-esteem are theoretically distinct constructs, differing in specificity and scope, they are closely related and both reflect students’ competence-related beliefs within the mathematics domain (Bandura, 1997; Marsh and Craven, 2006). While self-efficacy refers to task-specific judgments of capability, mathematics self-esteem captures a broader evaluative perception of competence and confidence in mathematics. Adolescents with higher mathematics self-esteem are therefore more likely to approach challenging problems confidently, persist despite setbacks, and achieve higher performance. Conversely, those with lower mathematics self-esteem may avoid mathematics tasks, experience heightened anxiety, and underperform academically.

SCT also highlights the importance of environmental influences, making it especially useful for examining differences between rural and urban educational contexts. For instance, smaller class sizes and closer teacher-student relationships in rural schools may give students more mastery experiences and social support, enhancing self-esteem and reducing anxiety. In contrast, highly competitive or resource-limited urban environments may increase stress and anxiety, potentially undermining students’ self-efficacy and performance. This dynamic interaction between the individual and the environment aligns closely with the present study’s exploration of how geographic location interacts with psychological factors to influence mathematics outcomes.

Together, CVT and SCT form a robust theoretical foundation for this research. While CVT explains how perceptions of control and value give rise to academic emotions such as mathematics anxiety, SCT provides insight into the development of self-esteem through environmental and social mechanisms. By integrating these frameworks, the study examines the direct effects of mathematics self-esteem and anxiety on achievement. It investigates how demographic factors such as gender, age, and location shape these processes. This theoretical integration allows for an in-depth interpretation of the findings: for example, an observed decline in self-esteem among older males may be understood through CVT as reflecting decreased perceived control during critical academic transitions, while SCT would frame this pattern as a consequence of reduced mastery experiences or negative social comparisons. Similarly, rural students’ heightened anxiety alongside stronger performance may be interpreted through the dual lenses of CVT and SCT, illustrating the complex interplay between emotional, cognitive, and contextual influences.

By grounding the study in these two complementary theories, the research moves beyond describing statistical associations to explaining why certain demographic groups experience different self-esteem, anxiety, and achievement patterns. This theoretical grounding also provides a basis for developing targeted interventions. For instance, strategies that increase students’ perceived control, such as mastery-based teaching approaches and constructive feedback, may reduce mathematics anxiety in line with CVT. At the same time, interventions informed by SCT, such as mentoring programs and supportive teacher-student relationships, can strengthen self-esteem and buffer against environmental stressors. Thus, combining CVT and SCT ensures that the study’s findings are interpreted within a comprehensive psychological framework that bridges theory, empirical evidence, and educational practice.

### Literature review

Adolescence is a key period in which students’ beliefs about their academic abilities, especially in mathematics, begin solidifying and strongly influencing their learning experiences and outcomes (Eccles and Wigfield, 2020). During these formative years, many students face increasing academic demands and social pressures, which can heighten both positive and negative emotions toward mathematics. Two psychological constructs have received considerable attention for their impact on mathematics performance: mathematics anxiety and mathematics self-esteem (often operationalised in the literature as domain-specific mathematics self-concept). Research consistently shows that these constructs play central roles in shaping students’ motivation, engagement, and achievement, making them critical factors to examine when addressing disparities in mathematics education (Barroso et al., 2021; Pekrun et al., 2017).

Mathematics anxiety is tension, worry, or fear that arises when individuals engage in mathematics tasks or anticipate mathematics-related situations (Ashcraft and Krause, 2007). It has cognitive and physiological components, often manifesting as racing thoughts, physical discomfort, and avoidance behaviours (Mosimege et al., 2024; Nzeadibe et al., 2023). High levels of mathematics anxiety interfere with working memory and problem-solving efficiency, which can directly undermine performance during assessments and class activities (Van Mier et al., 2019). Over time, anxiety may also lead students to avoid advanced mathematics courses, reduce their effort, and disengage from mathematics altogether, perpetuating a cycle of poor performance and increasing fear (Ramirez et al., 2018). Cross-national meta-analyses confirm a consistent negative relationship between mathematics anxiety and mathematics performance, with this effect appearing across diverse contexts such as the United States, China, Nigeria, and Finland (Barroso et al., 2021; Namkung et al., 2019).

In contrast, mathematics self-esteem reflects students’ confidence and beliefs in their ability to succeed in mathematics. It is closely tied to motivation, effort, and resilience in facing challenges. Students with higher self-esteem tend to set ambitious goals, use effective learning strategies, and persist when confronted with difficulties (Acosta-Gonzaga, 2023). Mathematics self-esteem also plays a crucial role in protecting students from the harmful effects of anxiety. Students who believe they are competent can better regulate negative emotions and maintain performance even under pressure. This buffering effect has been supported by recent studies conducted in diverse settings, including the U. S., South Africa, and China, showing that self-esteem moderates the relationship between mathematics anxiety and achievement (Liu et al., 2022; Rozgonjuk et al., 2020; Tshikovhele et al., 2024).

However, these relationships do not occur in isolation. Demographic factors, such as gender, age, and school context, influence how mathematics anxiety and self-esteem develop and interact. Gender differences are among the most well-documented findings. Large-scale international studies such as PISA have shown that girls consistently report higher levels of mathematics anxiety and lower mathematics self-esteem compared to boys, even when their performance is equivalent or higher (Goetz et al., 2013; Hill et al., 2016). These disparities have been linked to stereotype threat and societal expectations that frame mathematics as a male-dominated domain, which can undermine girls’ confidence and increase their fear of failure. Research across multiple regions has documented age-related effects on mathematics anxiety and self-esteem during adolescence. Studies indicate that mathematics anxiety tends to increase during middle adolescence (ages 14–17) as students transition into higher-stakes testing environments, while self-esteem may decline due to heightened social comparison and academic pressures (Dowker et al., 2016; Fan et al., 2019). Contextual influences, such as rural versus urban schooling, have been examined less frequently but are beginning to emerge in the literature. Research in China, Uganda, and Kenya has highlighted how rural schools often face challenges such as limited access to resources, fewer specialised teachers, and fewer opportunities for enrichment activities (Kloker et al., 2024; Makori and Onderi, 2014; Zhongzhuoma and Abdul Aziz, 2024). At the same time, rural schools may provide protective factors, including smaller class sizes and stronger teacher-student relationships, which can foster supportive learning environments and reduce anxiety (Arnold et al., 2005). These contrasts suggest that geographic location may interact with gender and age to shape mathematics-related beliefs and outcomes in complex ways.

An important insight from recent research is that mathematics anxiety and self-esteem interact dynamically. While anxiety typically predicts lower performance, its negative effects can be mitigated when students possess high levels of self-esteem. Conversely, when self-esteem is low, anxiety may have an even stronger detrimental impact on learning. For instance, experimental and longitudinal work shows that higher self-esteem and mastery/learning orientations buffer students against the disruptive effects of failure or negative feedback, whereas low self-esteem (and domain-contingent self-worth) increases vulnerability to performance declines under threat (Park et al., 2007; Niiya et al., 2004; Brown, 2010). Comparable country-level evidence has since appeared: South African university samples link self-esteem to student adjustment (Tshikovhele et al., 2024), Turkish adolescent data identify self-esteem and resilience as mediators of wellbeing (Turan et al., 2025), and a large longitudinal Chinese study finds that low self-esteem prospectively predicts higher anxiety across college years (Liu et al., 2022). Similarly, Rozgonjuk et al. (2020) found that mathematics self-efficacy significantly predicted mathematics anxiety among social sciences students in Estonia. These findings underscore the importance of considering both constructs together, rather than studying them in isolation, to better understand and address their impact on academic achievement.

Despite the rich body of research on mathematics anxiety and self-esteem, several important areas remain underexplored. Much of the existing literature has focused on Western, urban, and high-resource settings, leaving gaps in our understanding of how these psychological factors operate in diverse cultural and educational contexts. Fewer studies have examined how demographic factors such as gender, age, and geographic location interact to shape students’ experiences of anxiety and self-esteem. Furthermore, while previous research has provided valuable insights into the main effects of these variables, less attention has been paid to how self-esteem and anxiety work together to influence achievement. Understanding these interactions is essential for identifying the students most at risk and designing interventions that simultaneously address multiple sources of difficulty.

The present study builds on these gaps by exploring the complex relationships among mathematics anxiety, mathematics self-esteem, and mathematics achievement among adolescents. It focuses not only on the direct effects of anxiety and self-esteem but also on how these relationships are influenced by gender, age, and school context. By employing Bayesian beta regression, the study can capture the nuances of these interactions with greater precision, moving beyond traditional statistical approaches. This comprehensive analysis provides a deeper understanding of the psychological and contextual factors that shape mathematics achievement and offers insights that can inform targeted educational interventions. From this foundation, the study seeks to answer the following research question: How do mathematics self-esteem and mathematics/test anxiety relate to adolescents’ mathematics achievement, and how are these relationships influenced by gender, age, and geographic location (rural vs. urban)? To address this question, five hypotheses (H_0_) were formulated:

1. H_1_: Older male students (aged 17+) will exhibit lower mathematics self-esteem than younger and female peers.
2. H_2_: Rural students will report higher mathematics test anxiety than urban students.
3. H_3_: Despite higher anxiety, rural students will demonstrate higher mathematics achievement.
4. H_4_: Gender differences in mathematics achievement will be moderated by age, with males aged 15–16 showing higher achievement than other groups.
5. H_5_: Interactions among gender, age, and location will significantly explain variance in mathematics self-esteem, anxiety, and achievement.

## Methods

### Participants

The study included 329 adolescents recruited from secondary schools in urban and rural settings. Participants were categorised by gender (female: *n* = 158, male: *n* = 171), location (urban: *n* = 153, rural: *n* = 176), and age groups (13–14 years: *n* = 122, 15–16 years: *n* = 167, 17 years and above: *n* = 40). Inclusion criteria required enrollment in grades corresponding to the age groups and the ability to complete assessments in the administration language. Exclusion criteria included incomplete data or withdrawal. Recruitment occurred through schools during regular hours, with 400 adolescents approached and 350 consenting (87.5% response rate); 21 were excluded for missing data exceeding 20%. Informed assent was obtained from minors, with parental consent, and direct consent from those 18+. The sample was convenience-based, aiming to include diverse demographic backgrounds in mathematics education contexts.

### Measures

Data were collected using three standardised instruments to assess psychological and achievement aspects of mathematics. All scores ranged from 0 to 100, with higher values indicating greater levels of the construct.

### Mathematics self-esteem scale (MSE)

The MSE is a self-report questionnaire measuring students’ perceived competence and confidence in mathematics. It consists of 20 items rated on a 5-point Likert scale (1 = strongly disagree to 5 = strongly agree), with several items reverse-coded. Raw item scores were summed and linearly transformed to a 0–100 scale, with higher scores indicating higher mathematics self-esteem. In the present study, the MSE demonstrated high internal consistency (Cronbach’s *α* = 0.87; McDonald’s *ω* = 0.89). A confirmatory factor analysis supported a unidimensional structure with acceptable model fit indices (CFI = 0.92, RMSEA = 0.06), indicating that the scale reliably captured domain-specific mathematics self-esteem in this adolescent sample.

### Mathematics test anxiety inventory (MTAI)

The MTAI assesses anxiety related to mathematics testing. It includes 25 items on a 4-point scale (1 = almost never to 4 = almost always), summed and scaled to 0–100. Higher scores indicate elevated anxiety. The inventory showed strong consistency (*α >* 0.85) and validity in educational research (Hopko et al., 2003). Here, reliability was excellent (*α* = 0.88, *ω* = 0.90), with convergent validity evidenced by correlations with MSE and MAT.

### Mathematics achievement test (MAT)

The MAT evaluates mathematical knowledge and skills via 30 multiple-choice and short-answer questions covering algebra (40%), geometry (30%), and statistics (30%). Scores are percentage correct (0–100). The test was standardised for adolescents, ensuring content validity (Pekrun et al., 2002). Internal consistency in this sample was adequate (*α* = 0.82, *ω* = 0.84).

### Procedure

Participants completed the instruments in a single 45–60 min classroom session under supervision. The order was counterbalanced using a Latin square design at the individual level to minimise effects. No time limits applied to MSE/MTAI; MAT had a 45-min limit. Proctor-student ratios were approximately 1:20, with accommodations per school policy. Data were collected via coded IDs for linkage while maintaining anonymity. Responses were double-entered, cross-validated, and subjected to range/logic checks. Ethical approval details are noted in the Participants section.

### Data analysis

Analyses employed Bayesian beta regression to evaluate demographic effects (gender, location, age group) on MSE, MTAI, and MAT scores. Models were fitted in R (version 4.3.2) using the brms package (version 2.20.4) (Bürkner, 2017), interfacing with Stan (Stan Development Team, 2024) for MCMC sampling. This framework enables prior flexibility, uncertainty incorporation, and hierarchical regularisation. As no school identifiers were available, random intercepts were not included; hierarchical priors on fixed effects provided regularisation instead. Separate models were fitted for each outcome. Scores were rescaled to (0,1) by dividing by 100 and modelled with a beta distribution to accommodate bounds and prevent invalid predictions, which is suitable for continuous bounded social science data.

The model for rescaled outcome *Y* (e.g., MSE) for participant *i* is specified as a beta distribution (Equation 1):


(1) $$Y_{i}∼\text{Beta}({µ}_{i}\phi ,(1-{µ}_{i})\phi )$$

With the mean structure defined through a logit link function (Equation 2):


(2) $$\begin{array}{ll} 1\text{ogit}(\mathrm{\mu i}) & =\beta 0+\beta 1\cdot \text{Genderi}+\beta 2\cdot \text{Locationi}\\ & +\beta 3\cdot \mathrm{Age}15-16,i+\beta 4\cdot \mathrm{Age}17+,i\\ & +\beta 5\cdot \text{Genderi}\cdot \text{Locationi}\\ & +\beta 6\cdot \text{Genderi}\cdot \mathrm{Age}15-16,i\;(2)\\ & +\beta 7\cdot \text{Genderi}\cdot \mathrm{Age}17+,i\\ & +\beta 8\cdot \text{Locationi}\cdot \mathrm{Age}15-16,i\\ & +\beta 9\cdot \text{Locationi}\cdot \mathrm{Age}17+,i \end{array}$$

Predictors were dummy-coded: gender (0 = female, 1 = male), location (0 = urban, 1 = rural), and age group (reference: 13–14; dummies for 15–16, 17+). Models included main effects and two-way interactions. Priors were specified to regularise estimates, with Student-t distributions assigned to the regression coefficients (Equation 3) and the precision parameter (Equation 4).


(3) $$\beta_{j}\sim \text{Student}-t(3,0,2,5),j=0,\ldots 9$$

(4) $$\gamma_{0}\sim \text{Student}-t(3,0,2.5)$$

These were selected for social science applications (Gelman and Shalizi, 2013), with heavier tails for robustness and scaling for small effects. Prior predictive checks ensured plausible distributions. MCMC used 4 chains, 2000 iterations (1,000 warm-up), with convergence via *R*^ˆ^ *<* 1.01*, ESS >* 400. Fit is assessed by posterior predictive checks (PPCs) and a leave-one-out information criterion (LOOIC). Sensitivity tested alternative priors (e.g., wider scales).

## Results

In this section, we present the findings from our Bayesian hierarchical models, organised around the study’s five hypotheses (H_1_ – H_5_). This structure focuses on our key research question while integrating descriptive statistics, model estimates, and diagnostic checks. We start with an overview of the descriptive patterns to provide context, then address each hypothesis. All results are based on Beta regression models with a logit link, which account for the bounded nature of our outcomes (0–100 scores rescaled to 0–1). We report posterior means, standard errors (SE), 95% credible intervals (CI), and the posterior probability that the coefficient is positive [Pr(*β* > 0)] for model parameters. Bold entries in tables highlight effects where the 95% CI excludes zero. Throughout, we emphasise uncertainty: effects with CIs including zero are treated as suggestive, and even credible effects are interpreted cautiously given the logit scale’s baseline dependence.

Before presenting the results of the hypotheses, we summarise the descriptive statistics in Table 1. These groupwise means and standard deviations (SD) for mathematics self-esteem (MSE), mathematics test anxiety (MTAI), and mathematics achievement (MAT) offer a raw descriptive analysis of the data. MSE and MTAI hover near the scale midpoint (around 59 and 55, respectively) with moderate variability (SD ≈ 9–10). MAT scores are lower (mean ≈ 30) with a greater spread (SD ≈ 13), reflecting the test’s challenge for adolescents. Demographic differences appear subtle: males and females show similar averages; rural students score slightly higher on MSE, MTAI, and MAT than urban ones, while age groups are largely comparable. These patterns set the stage for our models, which adjust for interactions and incorporate priors to regularise estimates.

**Table 1:** Descriptive statistics for MSE, MTAI, and MAT scores by demographic variables.

Variable	Category	MSE	MTAI	MAT
		Mean (SD)	Mean (SD)	Mean (SD)
Gender	Female (*n* = 158)	59.4 (8.93)	55.4 (10.08)	29.0 (12.49)
	Male (*n* = 171)	58.6 (8.60)	55.0 (9.87)	30.3 (14.16)
Location	Urban (*n* = 153)	57.8 (8.76)	53.9 (9.95)	29.0 (13.83)
	Rural (*n* = 176)	60.0 (8.70)	56.3 (9.91)	30.2 (13.02)
Age Group	13–14 years (*n* = 122)	59.0 (8.71)	56.2 (9.54)	28.3 (12.60)
	15–16 years (*n* = 167)	59.0 (8.71)	54.8 (10.37)	30.4 (14.44)
	17 + years (*n* = 40)	59.1 (9.44)	55.3 (9.04)	28.1 (10.56)
Overall	(*N* = 329)	59.0 (8.76)	55.2 (9.97)	29.7 (13.40)

Table 2 displays the posterior summaries for all parameters across the three outcomes. These come from separate MSE, MTAI, and MAT models, each including main effects and two-way interactions for gender, location, and age. The Student-*t*(3, 0, 2.5) priors help prevent overfitting, and diagnostics (e.g., *R*^ˆ^ *<* 1.01, ESS *>* 400) confirm reliable sampling. Posterior predictive checks show the Beta distribution fits the data well, capturing central tendencies and variability without major discrepancies.

**Table 2:** Parameter estimates from Bayesian beta regression models.

Outcome	Parameter	Estimate	SE	95% CI		Pr(β > 0)
				Lower	Upper	
	Intercept	0.320	0.059	0.201	0.434	1.000
	Gender	0.076	0.077	−0.080	0.222	0.830
	Location	0.105	0.080	−0.050	0.263	0.907
	Age 15–16	−0.008	0.081	−0.166	0.150	0.460
MSE	Age 17+	0.053	0.097	−0.139	0.242	0.706
	Gender × Location	−0.106	0.085	−0.271	0.064	0.105
	Gender × Age 15–16	−0.073	0.088	−0.250	0.093	0.209
	Gender × Age 17+	−**0.344**	0.135	−**0.608**	−**0.079**	**0.007**
	Location × Age 15–16	0.020	0.091	−0.161	0.195	0.594
	Location × Age 17+	0.149	0.145	−0.126	0.440	0.856
	Intercept	0.152	0.065	0.025	0.280	0.990
	Gender	0.024	0.088	−0.148	0.198	0.609
	Location	0.140	0.084	−0.023	0.306	0.954
	Age 15–16	0.048	0.091	−0.132	0.224	0.698
MTAI	Age 17+	0.053	0.113	−0.166	0.277	0.681
	Gender × Location	0.067	0.094	−0.111	0.256	0.754
	Gender × Age 15–16	−0.111	0.100	−0.308	0.087	0.129
	Gender × Age 17+	−0.081	0.154	−0.381	0.223	0.301
	Location × Age 15–16	−0.121	0.096	−0.309	0.068	0.104
	Location × Age 17+	−0.117	0.104	−0.420	0.188	0.226
	Intercept	−0.854	0.117	−1.089	−0.631	0.000
	Gender	−0.067	0.149	−0.362	0.221	0.333
	Location	0.126	0.149	−0.167	0.418	0.802
	Age 15–16	−0.003	0.155	−0.305	0.303	0.489
MAT	Age 17+	0.164	0.194	−0.224	0.537	0.808
	Gender × Location	−0.135	0.163	−0.459	0.180	0.197
	Gender × Age 15–16	**0.471**	0.172	**0.130**	**0.803**	**0.997**
	Gender × Age 17+	−0.184	0.259	−0.706	0.311	0.247
	Location × Age 15–16	−0.157	0.163	−0.482	0.158	0.163
	Location × Age 17+	−0.169	0.277	−0.720	0.364	0.274

Bold entries indicate effects with 95% credible intervals excluding zero. Pr(β > 0) is the posterior probability that the coefficient is positive; for negative effects, Pr(β < 0) = 1 − Pr(β > 0). Models fitted using Bayesian Beta regression with Student-t(3, 0, 2.5) priors. MSE, Mathematics Self-Esteem; MTAI, Mathematics Test Anxiety Inventory; MAT, Mathematics Achievement Test.

For H_1_, we predicted that older male students (aged 17+) would have lower mathematics self-esteem than younger or female peers. The model supports this: the gender × age (17+) interaction for MSE is negative (posterior mean = − 0.344, 95% CI [− 0.608, − 0.079], Pr(β > 0) = 0.007). This suggests that, after accounting for other factors, older males’ expected MSE is lower than the sum of main effects would imply. This interaction points to a meaningful dip in self-esteem for this group on the logit scale, though the exact shift on the 0–100 scale varies with baseline levels (around 59 in our data). Main effects for gender, location, and age are small, with CIs including zero, highlighting that the effect emerges through interaction. Descriptives in Table 1 show flat averages, but the model teases out this nuanced pattern by pooling information across groups.

**Figure 1:**
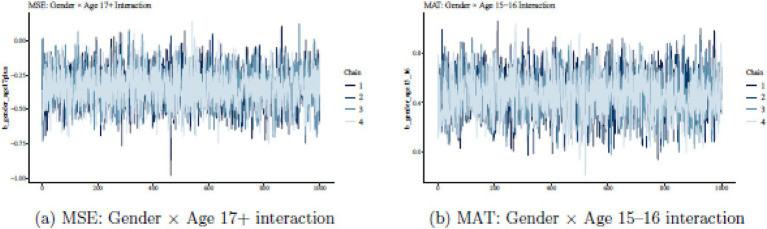
Trace plots for gender × age interaction parameters. Trace plots display MCMC convergence across four chains for the significant interaction parameters. Both parameters demonstrate stable mixing, with no evidence of poor convergence or divergent transitions.

**Figure 2:**
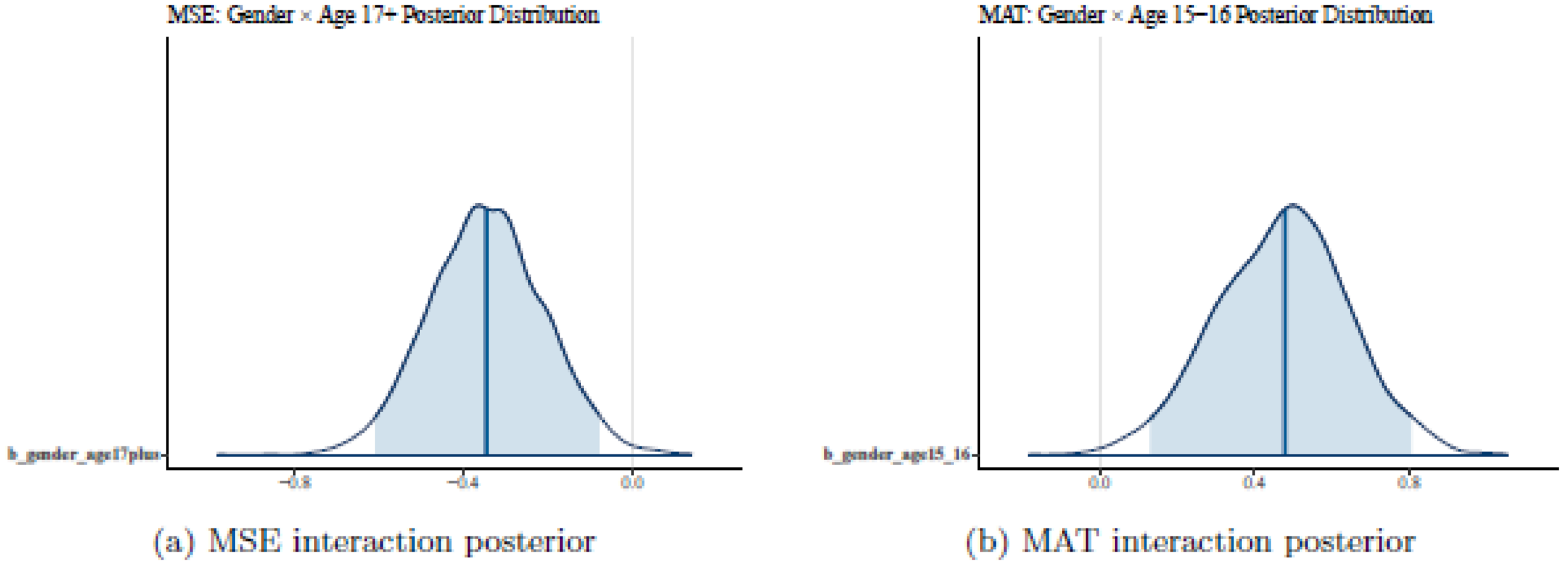
Posterior distributions for significant gender × age interactions. The MSE gender × age 17 + effect shows a clearly negative posterior distribution (left), while the MAT gender × age 15–16 effect shows a clearly positive distribution (right). Both 95% credible intervals exclude zero, indicating statistically meaningful effects.

To assess the reliability of this H_1_ finding, we examined MCMC diagnostics. Figure 1A shows trace plots for the interaction term: chains mix well, with no drift or sticking. The posterior density in Figure 2A is unimodal and shifted to the left of zero, confirming the negative effect’s credibility. Sensitivity analyses with different prior scales yielded similar results, and posterior predictive checks matched the observed MSE distribution. Thus, the evidence favours H_1_, indicating a specific vulnerability in older males’ mathematics self-esteem.

H_4_ proposed that gender differences in mathematics achievement would be moderated by age, with males aged 15–16 showing higher scores. The data align with this: the gender × age (15–16) interaction for MAT is positive (mean = 0.471, 95% CI [0.130, 0.803], PD = 0.997). This indicates an achievement boost for mid-adolescent males beyond additive effects. Like H_1_, main effects are inconclusive (CIs include zero), emphasising the interaction’s role. Descriptives show a slight edge for 15–16-year-olds, but the model pins it to males.

Diagnostics reinforce this: Figure 1B displays smooth chains, and Figure 2B shows a right-shifted density. Prior sensitivity and predictive checks were satisfactory, supporting H_4_ as a credible developmental peak in male achievement. Comparing H_1_ and H_4_ reveals an intriguing pattern: mid-adolescent males excel in achievement (H_4_), but older ones lag in self-esteem (H_1_). This asynchrony fits social-cognitive theories, where early successes may not sustain confidence amid later pressures like transitions. Since models are separate, we avoid causal claims, but the contrast invites further exploration.

For H_2_, we expected rural students to have higher mathematics test anxiety than urban ones. The location main effect for MTAI is positive (mean = 0.140, 95% CI [−0.023, 0.306], PD = 0.954), suggesting elevated anxiety in rural areas. However, the CI brushes zero, so the evidence is suggestive, not decisive. Descriptives confirm a small rural increase (56.3 vs. 53.9). Interactions with age or gender have CIs including zero, implying a broad rather than subgroup-specific effect. MCMC traces and densities (not shown for MTAI interactions, but overall diagnostics are strong) support this tentative finding. Sensitivity checks keep the sign positive, though uncertainty persists. Thus, H_2_ has qualified support, warranting low-risk interventions like rural test-prep programs.

H_3_ anticipated that rural students would show higher achievement despite elevated anxiety. Results are less clear: the rural main effect for MAT is positive (mean = 0.126, 95% CI [−0.167, 0.418]), but uncertain (includes zero). For MSE, it is similar (mean = 0.105, 95% CI [−0.050, 0.263]). Descriptives favour rural groups modestly, but regularisation shrinks after adjustment. No location interactions exclude zero, suggesting diffuse effects. Given H_2_’s qualified rural anxiety increase, the proposed tradeoff is not strongly evident. Diagnostics are solid, but larger samples or multilevel data might be clarified. H_3_ thus receives tentative backing: rural advantages in achievement and self-esteem seem possible but small.

H_5_ posited that interactions among gender, age, and location would explain significant variance across outcomes. Support is mixed but positive overall. Leave-one-out cross-validation (LOO-IC) prefers interaction models over main-effects ones for MSE (gain ≈ 8.4) and MAT (≈ 7.7), but not MTAI. This aligns with credible interactions for MSE (H_1_) and MAT (H_4_), while MTAI shows mainly the location effect. Figures 1, 2 illustrate well-behaved posteriors for key interactions, bolstering H_5_. Interactions add value where demographics intersect meaningfully, as in developmental shifts for self-esteem and achievement.

Given the foregoing, the use of the Bayesian approach uncovers critical patterns: credible gender–age interactions for MSE (H_1_) and MAT (H_4_), suggestive rural effects for MTAI (H_2_) and MAT/MSE (H_3_), and overall benefits from including interactions (H_5_). These findings, grounded in robust estimates and diagnostics, highlight demographic influences on mathematics outcomes while acknowledging uncertainty. Descriptives provide context, but models reveal interactions obscured by raw means. This balanced view informs targeted educational strategies without overclaiming.

## Discussion

The study examined how gender, age, and geographic location influence adolescents’ mathematics self-esteem, test anxiety, and achievement. Using Bayesian beta regression, this research revealed in-depth patterns beyond traditional main-effect analyses, offering a deeper understanding of the psychological and contextual factors influencing mathematics learning and performance. The findings are interpreted here in light of the study’s hypotheses, previous research, and the CVT and SCT theoretical frameworks.

As predicted in H_1_, older male students (aged 17 and above) exhibited significantly lower mathematics self-esteem than younger males and females. This decline likely reflects the heightened academic demands and social pressures of late adolescence, when students face high-stakes examinations and decisions about future education or careers. Such transitions often provoke increased peer comparison and heightened sensitivity to failure, leading to erosion of confidence in mathematical ability. This finding is consistent with previous research by Dowker et al. (2016) and Fan et al. (2019), who reported declines in self-esteem as adolescents progress through increasingly competitive academic environments. CVT provides a useful lens here: low self-esteem may emerge when students place a high value on success in mathematics but perceive little control over their ability to achieve desired outcomes (Pekrun et al., 2017). SCT complements this interpretation by emphasising environmental influences, suggesting that older males may experience fewer mastery experiences and less supportive teacher feedback, reducing their self-efficacy. These results highlight the need for early interventions, during mid-adolescence, to build and sustain mathematical confidence before it begins to decline in later years.

Although prior research frequently reports higher mathematics self-esteem among males across age groups, the lower self-esteem observed among older males in this study may reflect developmental and contextual pressures specific to late adolescence. As students approach the end of secondary schooling, academic evaluation becomes more consequential and performance expectations intensify, particularly in systems characterised by high-stakes examinations (Eccles and Wigfield, 2020; Dowker et al., 2016). Under such conditions, repeated academic challenges or heightened performance pressure may undermine previously stable confidence, even among students who earlier exhibited higher self-beliefs. From a Control-Value Theory perspective, this pattern suggests that older males may continue to place high value on mathematics outcomes while experiencing reduced perceived control, resulting in diminished mathematics self-esteem (Pekrun et al., 2017). Social Cognitive Theory further suggests that reduced mastery experiences, intensified social comparison, and evaluative feedback during this transitional period may erode confidence despite earlier advantages (Bandura, 1997).

Turning to H_2_, rural students were expected to report higher levels of mathematics test anxiety than urban students. The results provided partial support for this hypothesis, with rural students showing slightly elevated anxiety, though the effect was not strong. This pattern may stem from rural schools’ structural challenges, such as limited access to specialised mathematics teachers, outdated resources, and fewer enrichment opportunities (Makori and Onderi, 2014; Maniram, 2023). These barriers can leave students feeling underprepared for high-stakes testing, increasing evaluative stress. Similar findings have been reported in Uganda and China, where rural students’ anxiety was linked to systemic disadvantages (Kloker et al., 2024; Zhongzhuoma and Abdul Aziz, 2024). From a CVT perspective, elevated anxiety arises when students value mathematics highly as a pathway to future success but perceive limited control over their performance. SCT adds that rural students may lack role models or positive peer comparisons, weakening their self-efficacy and further intensifying anxiety. Although modest, this trend suggests that rural learners would benefit from targeted test preparation and anxiety-reduction interventions designed to enhance their perceived control.

Interestingly, H_3_ proposed that rural students, despite experiencing higher anxiety, would demonstrate higher mathematics achievement. This hypothesis received partial support, as rural students displayed slightly higher achievement and self-esteem than their urban peers, though these effects were small and uncertain. This apparent paradox suggests that rural contexts embody both risk and protective factors. While resource scarcity can heighten anxiety, rural schools often foster close teacher-student relationships and strong community ties, which promote emotional support and individualised attention. These factors may help explain why rural students can achieve well despite structural disadvantages, echoing findings by Arnold et al. (2005) and Batiibwe et al. (2020). SCT provides insight into this dynamic: positive reinforcement and social persuasion in rural environments can strengthen students’ self-efficacy, encouraging persistence despite challenges. CVT suggests that these students may feel a sense of control within their immediate environment, buffering the negative effects of broader systemic barriers. However, the co-occurrence of higher anxiety and modest achievement indicates that this perceived control is fragile, especially during high-stakes assessments.

The role of developmental timing was further highlighted in H_4_, which predicted that mid-adolescent males (ages 15–16) would show higher mathematics achievement than other groups. This hypothesis was strongly supported. The higher performance of mid-adolescent males likely reflects a convergence of cognitive maturation and motivational factors. At this stage, students develop advanced problem-solving skills while benefiting from intrinsic motivation and external encouragement, particularly in contexts where mathematics is culturally valued. This result aligns with Larsen and Luna (2018), who identified mid-adolescence as a critical period for higher-order cognitive development. Interpreted through CVT, these students likely perceive strong control over their performance and high value in mathematics, fostering positive emotions such as pride and enjoyment. SCT reinforces this view by highlighting the role of mastery experiences and supportive social networks in sustaining engagement. However, this advantage may fade without deliberate efforts to maintain motivation and confidence, as seen in the decline in self-esteem among older males discussed earlier under H_1_.

Finally, H5 anticipated that interactions among demographic variables would explain meaningful variance in mathematics self-esteem, anxiety, and achievement. This hypothesis received partial support, as the inclusion of two-way interaction terms improved model fit for mathematics self-esteem and achievement, but not for test anxiety. Specifically, interactions between gender and age were particularly informative, revealing distinct developmental patterns that were not apparent in main-effect models alone. These findings underscore the importance of examining how demographic characteristics combine pairwise, rather than in isolation, to shape students’ mathematics-related outcomes. For example, the observed interaction between gender and age highlights that developmental changes in self-esteem and achievement differ for male and female students across adolescence. Similarly, interactions involving geographic location suggest that contextual influences may modify demographic effects, even when such effects are modest. Interpreted through the Control-Value Theory, these interaction effects reflect shifts in perceived control and value that vary across demographic subgroups, producing different emotional and motivational responses to mathematics. Social Cognitive Theory further suggests that these patterns arise from differences in mastery experiences and social reinforcement across demographic contexts. Together, these results demonstrate that two-way demographic interactions meaningfully enhance explanatory power, even in the absence of higher-order interaction effects.

## Conclusion

This study revealed how gender, age, and geographic location influence adolescents’ mathematics self-esteem, test anxiety, and achievement. Using Bayesian beta regression, we uncovered complex and interrelated patterns that extend beyond the limitations of traditional statistical approaches. The most salient findings include a marked decline in self-esteem among older male students despite earlier achievement advantages, a developmental peak in achievement among mid-adolescent males, and the paradoxical pattern of rural students displaying slightly higher achievement and elevated test anxiety. These results underscore that psychological and academic outcomes in mathematics are not driven by single factors, but by the dynamic interplay of developmental stage, social identity, and learning context.

The strength of this study lies in its innovative methodological approach and theoretical integration. By applying Bayesian beta regression, we accounted for uncertainty and identified subtle demographic interactions that would likely remain hidden in conventional analyses. Grounding the findings in Control-Value Theory and Social Cognitive Theory provided a comprehensive explanatory framework, linking individual perceptions of control and self-efficacy to broader environmental influences. This dual focus enabled a deeper understanding of why certain groups, such as older rural males, are particularly vulnerable, while others demonstrate resilience despite structural challenges.

Collectively, these insights challenge simplistic urban-centric or gender-generalised narratives and highlight the need for developmentally informed, context-sensitive interventions. By illuminating risk factors and protective mechanisms, this research offers a roadmap for educators and policymakers to design strategies that simultaneously reduce mathematics anxiety, strengthen self-esteem, and enhance achievement. Ultimately, this study advances the conversation on equity in mathematics education by demonstrating that meaningful progress depends on recognising and responding to the intricate intersections of demographic and psychological factors that shape adolescents’ learning trajectories.

### Educational implications

The findings of this study have implications for a range of stakeholders, including teachers, school leaders, policymakers, and researchers concerned with improving mathematics education and adolescent wellbeing. The observed decline in self-esteem among older male students suggests that educators and school psychologists would benefit from greater awareness of the critical period between mid- and late adolescence. Early identification of students at risk of declining confidence could inform targeted support initiatives, helping to sustain motivation as academic demands increase. Such initiatives would likely focus on fostering mastery experiences, constructive feedback, and positive peer interactions, which are central to building perceived control and self-efficacy according to CVT and SCT.

The higher mathematics achievement among mid-adolescent males implies that this developmental stage represents a window of opportunity for strengthening performance and psychological resilience. Teachers and curriculum planners might consider capitalising on this period by reinforcing positive academic emotions and helping students view fluctuations in confidence as a normal part of learning. This would help maintain motivation and prevent the subsequent declines in self-esteem observed among older students.

In rural contexts, the combination of slightly higher achievement and elevated test anxiety highlights a complex balance of risk and resilience. Educational leaders and policymakers may need to recognise that rural schools often cultivate strong relationships and community support that promote confidence. However, structural challenges such as limited resources and exposure to diverse assessment practices could contribute to evaluative anxiety. Addressing these challenges would likely involve strengthening access to varied assessment experiences and support structures that enhance students’ sense of control over performance.

Finally, the significant interactions among gender, age, and location emphasise the need for context-sensitive educational policies. One-size-fits-all approaches to mathematics education are unlikely to meet the needs of diverse learners. Teacher professional development and resource allocation strategies would benefit from considering how demographic factors intersect to shape students’ psychological and academic experiences. Recognising these intersections could enable more equitable interventions, ensuring that support reaches those most at risk of disengagement or underperformance.

### Limitations and future directions

This study has several limitations. Using a convenience sample limits the generalizability of the findings, and the cross-sectional design prevents conclusions about developmental changes or causality. In addition, the lack of school-level data, such as teacher quality or resource availability, restricted the examination of contextual influences. Although Bayesian beta regression provided robust estimation, results may be sensitive to prior specifications and warrant replication with larger, more diverse samples. Another limitation is that the study did not include a direct measure of mathematics anxiety as a domain-specific construct distinct from test anxiety. Although test anxiety captures evaluative stress associated with examinations, mathematics anxiety more broadly reflects fear and tension related to engaging with mathematical content and has been consistently shown to be negatively associated with mathematics achievement. While mathematics anxiety is related to mathematics self-esteem and test anxiety, it represents a theoretically distinct construct. Future research should therefore incorporate direct measures of mathematics anxiety alongside test anxiety and self-esteem to more fully disentangle their unique and joint contributions to mathematics achievement. Future studies could also adopt longitudinal and multilevel designs to track changes over time and capture the broader educational context. Mixed-methods and intervention-based research would further deepen understanding and test strategies to reduce anxiety, strengthen self-beliefs, and enhance achievement among vulnerable subgroups, particularly older males and rural learners.

### Recommendations

Based on the findings of this study, several actions should be taken to strengthen mathematics education and support adolescent learners’ psychological wellbeing.

Early identification of at-risk students is essential. Schools and educational authorities should establish systems to monitor self-esteem levels, particularly among older male learners, to detect declines before they become entrenched. Targeted interventions at this stage would help sustain motivation and confidence as students transition into higher-stakes academic settings.

Addressing rural test anxiety is another critical priority. Efforts should focus on reducing evaluative pressures through access to diverse assessment formats, supportive testing environments, and increased exposure to test preparation strategies. At the same time, policymakers should invest in strengthening educational resources in rural communities while preserving the close teacher-student relationships and community support structures that foster resilience and persistence.

Teacher professional development must adapt to demographic realities. Training programs should equip educators to recognise how gender, age, and geographic context intersect to influence mathematics achievement and psychological outcomes. Mathematics education policies should embrace an intersectional approach, tailoring interventions to meet the needs of specific subgroups rather than applying uniform solutions.

Finally, research efforts must continue to build an evidence base for practice. Longitudinal and intervention-based studies should be undertaken to evaluate strategies aimed at reducing anxiety, fostering self-esteem, and improving achievement among vulnerable groups, particularly older males and rural learners. Such research will provide the empirical foundation to guide sustainable, equity-focused reforms.

## Data Availability

The raw data supporting the conclusions of this article will be made available by the authors, without undue reservation.
